# A Pair of Compound Heterozygous *IARS2* Variants Manifesting West Syndrome and Electrolyte Disorders in a Chinese Patient

**DOI:** 10.1055/s-0043-1778091

**Published:** 2024-01-16

**Authors:** Feiyu Zhou, Gui Yi, Xiangyu Liu, Wenchao Sheng, Jianbo Shu, Dong Li, Chunquan Cai

**Affiliations:** 1 Tianjin Children's Hospital (Tianjin University Children's Hospital), Tianjin, People's Republic of China; 2 Graduate College of Tianjin Medical University, Tianjin Medical University, Tianjin, People's Republic of China; 3 Department of Neurology, Tianjin Children's Hospital (Tianjin University Children's Hospital), Tianjin, People's Republic of China; 4 Tianjin Pediatric Research Institute, Tianjin Children's Hospital (Tianjin University Children's Hospital), Tianjin, People's Republic of China; 5 Tianjin Key Laboratory of Birth Defects for Prevention and Treatment, Tianjin Children's Hospital, Tianjin, Peoples' Republic of China

**Keywords:** West syndrome, CAGSSS, aminoacyl-tRNA synthetases

## Abstract

**Background** Aminoacyl-tRNA synthetases (ARSs) are evolutionarily conserved enzymes that ensure the accuracy of the translation process. Isoleucyl-tRNA synthetase 2 ( *IARS2* ) gene is a type of ARS that encodes mitochondrial isoleucine-tRNA synthetase. Pathogenic variants in the *IARS2* gene are associated with mitochondrial disease which involves several patients presenting broad clinical phenotypes. These clinical phenotypes include West syndrome, Leigh syndrome, and Cataract, growth hormone deficiency, sensory neuropathy, sensorineural hearing loss, and skeletal dysplasia syndrome. Only 29 cases have been reported worldwide. The patient manifested recurrent convulsions, and specific clinical manifestations included electrolyte disorders and recurrent infections.

**Methods** Whole-exome sequencing was performed on the child with West syndrome. Three-dimensional structure reconstruction and thermodynamic stability prediction were performed to further analyze the relationship between variation and phenotype.

**Conclusion** This study further expands the clinical spectrum of *IARS2* pathogenic variants. The case summaries help raise clinical awareness of *IARS2* -associated disease and reduce misdiagnosis.

**Result** In this report, a 13-month-old girl was diagnosed with West syndrome and Leigh syndrome for 7 months. Compound heterozygous variants in the IARS2 gene (NM_018060.4), c.2450G>A (Arg817His) and copy number variation (NC_000001. 11: g. (220267549_220284289) del), were detected by WES. This study further expands the clinical spectrum of IARS2 pathogenic variants. The case summaries help raise clinical awareness of IARS2-associated disease and reduce misdiagnosis.

## Introduction

Aminoacyl-tRNA synthetases (ARSs) are a family of enzymes that fulfill an integral step in the initiation of translation. 1 Protein synthesis is dependent on the selectivity of ARSs to recognize specific amino acids and the correct transfer RNAs (tRNAs). 2 Translation occurs at two different locations, including cytoplasm and mitochondria. Therefore, ARSs are divided into two types, ARS1 and ARS2. Eighteen ARSs act only in cytoplasmic lysate (ARS1) and 17 act only in mitochondria (ARS2). Variants in different genes encoding mitochondrial ARSs lead to phenotypes that are specific to tissues. 3 Neurological disease is associated with variants of five ARS genes ( *IARS2* , *DARS2* , *RARS2* , *FARS2* , *EARS2* ). 4 5 It is noteworthy that Isoleucyl-tRNA synthetase 2 ( *IARS2* ) gene is an important member of ARS family. *IARS2* gene, located at 1q41, spans approximately 53.9 kb and contains 23 exons. It encodes isoleucine-tRNA synthetase, a type I mitochondrial ARS. 6 *IARS2* gene is expressed in many tissues such as kidney, adrenal, brain, and thyroid and these tissues expression is similar. 7 The *IARS2* gene variants are related to West syndrome, Cataract, growth hormone deficiency, sensory neuropathy, sensorineural hearing loss, and skeletal dysplasia syndrome (CAGSSS), and Leigh syndrome. Both CAGSSS (OMIM: 616007) and Leigh syndrome (OMIM: 256000) are autosomal recessive. 5

West syndrome is an epileptic syndrome characterized by spastic seizures, characteristic electroencephalography (EEG), and intellectual disability. It is highly heterogeneous and has a complex etiology, with over 200 reported cases. 6 West syndrome is characterized clinically by clusters of spastic seizures (ES), high EEG dysrhythmias, and psychomotor developmental regression.

We report a pair of compound heterozygous IARS2 variants manifesting West syndrome with electrolyte disorders and recurrent infections in a Chinese patient. This case expands the clinical spectrum of IARS2 pathogenic variants.

## Case Presentation

The proband is a 14-month-old Asian female with a birth weight of 3,190 g born to non-consanguineous parents. She started to hold head up at the age of 4 months. The patient raised her head at 4 months of age and raised her head steadily at 8 months of age. At 6 months of age, she would eat her hands, followed sounds with her eyes, and occasionally she could pronounce vowels such as “a, o.” At 1 year of age, the patient was unsteady with a firm neck, could not roll over, could not grasp objects, could not pronounce consonants, and did not make eye contact.

**Fig. 1:**
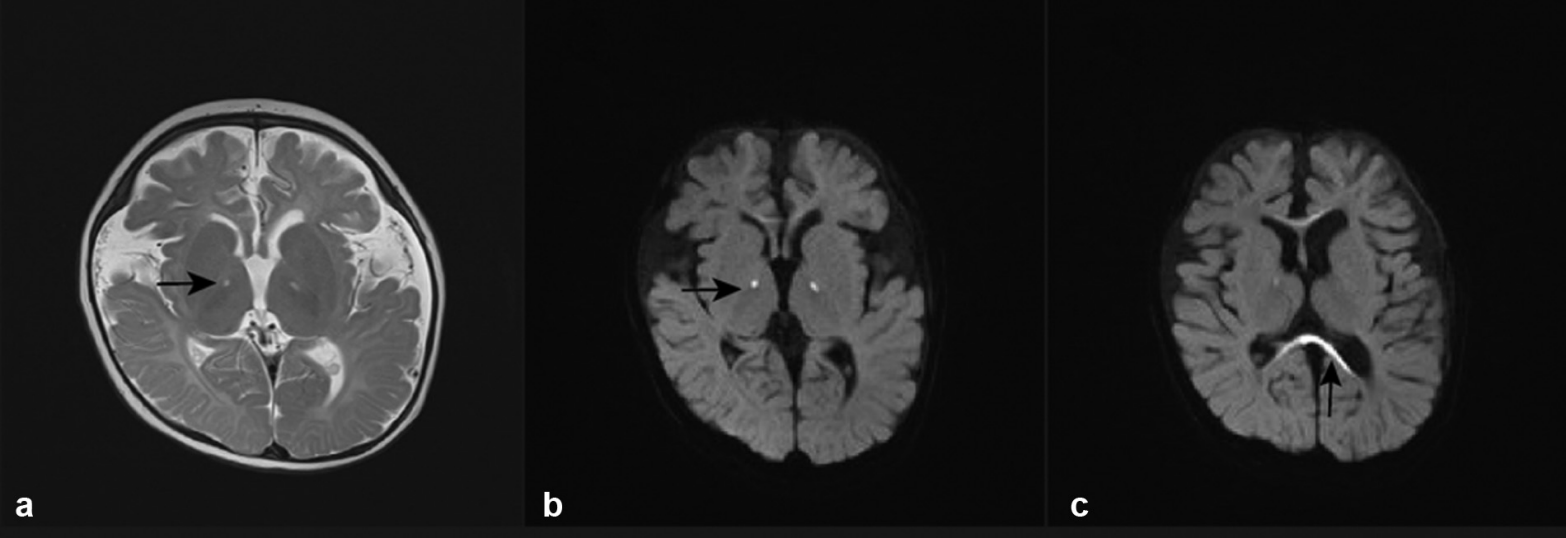
Brain magnetic resonance imaging. **(a)** A symmetric point-like lamellar high signal in the posterior limb of the internal capsule and widened ventricles and extracerebral gaps on T2WI sequences. **(b)** A symmetric point-like lamellar high signal in the posterior limb of the internal capsule on DWI sequences. **(c)** High signal in corpus callosum bilaterally on DWI sequences.

At 6 months of age, the patient developed infantile spasm. Laboratory studies showed elevated serum lactic acid at 9.4 mmol/L (reference range 0.5–2.2) and hypocalcemia at 1.52 mmol/L (reference range 2.10–2.80). Video EEG showed that two suspicious seizures were monitored during the waking period, manifesting as a slight upward lift of both upper limbs, with extensive medium–high amplitude slow waves compounded by low–moderate amplitude fast waves. The patient had significant motor backwardness. Therefore, electromyography (EMG) was done to learn whether there was any muscle or peripheral nerve damage. The EMG showed electromyographic activity in both upper limbs during the same period. The child was diagnosed with West syndrome and evaluated for global developmental delay on Developmental Quotient Assessment (Developmental Quotient Assessment: Adaptability 39, Gross Motor 41, Fine Motor 44, Language 31, Personal–Social 52). Head magnetic resonance imaging (MRI) showed that a symmetric point-like lamellar high signal in the posterior limb of the internal capsule and corpus callosum bilaterally on T2-weighted imaging (T2WI) and DWI sequences, long T1 and long T2 signal in the left temporal vault, small corpus callosum, widened ventricles, and extracerebral gaps ( Fig. 1 ). These MRI findings were consistent with Leigh syndrome. Echocardiography showed left ventricular wall thickening and tricuspid regurgitation.

The patient had multiple episodes of infantile spasm, and consequently developed intractable epilepsy. The patient was found to have visual impairment and left ventricular hypertrophy when she was 11 months old. In addition, the patient was diagnosed with anemia and multiple infections during several hospitalizations. She was initially treated with high-dose vitamin B6 and adrenocorticotropic hormone (ACTH) antiepileptic during hospitalization. The convulsions gradually reduced, and the reexamination of EEG showed improvement. The child was treated with Topamax after discharging from the hospital to control convulsions.

The patient's last seizure was when she was 13 months old, with most of the seizures occurring 3 to 5 minutes after waking up from sleep. Examination at age 13 months revealed following growth parameters: weight 11.8 kg (+2SD), height 75 cm, and head circumference 43 cm (−2SD). Except for a slowing of background activity, the final EEG showed no significant change from the previous one. Hypocalcemia (1.73 mmol/L, reference range 2.10–2.80), hyperlactatemia (3.82 mmol/L, reference range 0.5–2.2), and hypoparathyroidism (0.26 pmol/L, reference range 1.6–6.9) were detected. The child was treated with vitamin B6, valproic acid, and ACTH as before, but only showed transient effects. She developed intractable epilepsy. In addition, the patient was diagnosed with anemia and multiple infections and electrolyte disorders during several hospitalizations.

DNA extraction from peripheral blood samples was performed by the Tianjin Children's Hospital (Tianjin, China) in August 2022. The assay was performed on the Illumina sequencing platform which was built and validated by Goldcorp Medical. The whole-exome sequencing showed a compound heterozygous pathogenic variant, c.2450G > A (p. Arg817His) inherited from mother and copy number variation (CNV) (NC_000001. 11: g. [220267549_220284289] del) in the *IARS2.* The variant has been reported in the literature in patients with CAGSSS, Leigh syndrome, and West syndrome. 5 An approximately 16.7-kb deletion occurred in the chr1q41 region, involving exons 1 to 11 of *IARS2* from her father. According to the classification of gene variation by American College of Medical Genetics, the variant could be classified as of uncertain significance. Compared with literature reports published, the CNV variant is a novel variant, and the clinical phenotype of the patient is consistent with the published cases.

## Discussion

This article reports a case of West syndrome and Leigh syndrome caused by compound heterozygous variants in the *IARS2* gene, c.2450G > A (p. Arg817His) and CNV (NC_000001. 11: g. [220267549_220284289] del), presenting with West syndrome, developmental delay, and electrolyte disorders. *IARS2* is a type of ARS that encodes mitochondrial isoleucine-tRNA synthetase. IARS2 included two domains (HIGH and KMSKS). HIGH domain was destroyed due to 16.7-kb deletion which may have serious implications for protein structure and function.

ARSs are evolutionarily conserved enzymes that catalyze the attachment of amino acids to their cognate tRNAs, ensuring the accuracy of the translation process. Pathogenic ARS gene variants cause phenotypes in tissues with high metabolic demand. 8 Mitochondrial diseases can involve multiple organs and systems. Patients with the *IARS2* variant manifested neurological and muscular lesions. In our case, the central nervous system symptoms are more pronounced. The mechanism by which *IARS2* gene variants lead to tissue phenotypes is still not clear.

There are no gender and ethnic difference among the 29 cases with *IARS2* gene variants. Mitochondrial gene variants lead to diseases that can be multisystemic. 9 It is particularly pronounced in the nervous system because of the high-energy demand of nerve cells. 10 Only a small number of patients with *IARS2* -related disorders have been reported with West syndrome (4/29). Children were diagnosed with West syndrome as early as 5 months of age and as late as 10 months of age. 5 9 Leigh syndrome is mostly diagnosed within 1 year of age. In the reported cases related with *IARS2* , 10 cases (10/29) were diagnosed with Leigh syndrome. 5 8 9 11 Most of patients present with convulsions within 1 year of age.

Mitochondrial diseases are associated with endocrine dysfunction. 12 One of the most obvious symptoms of CAGSSS is short stature because *IARS2* variants are closely associated with growth hormone deficiency. However, children with short stature account for only a fraction of children with the IARS2 variant (13/29). As a result, normal stature does not rule out the possibility of IARS2 variants in mitochondrial diseases. Endocrine disorders in patients with hereditary mitochondrial disease may include diabetes, growth hormone deficiency, hypogonadism, adrenal dysfunction, hypoparathyroidism, and thyroid disease. 12 This study and four other studies have reported a total of five patients with hypoparathyroidism related with *IARS2* variants, which expands the spectrum of endocrine system clinical manifestations. 8 13 14 Seizures may be related to hypoparathyroidism. 15

The *IARS2* variant may be related to the disruption of the respiratory chain of erythrocytes leading to erythrocytopenia in this child. 13 Patients with *IARS2* gene variants reported in the previous literature mostly had neurological and endocrine symptoms. In addition, the proband has multiple respiratory and gastrointestinal infections after birth. No relevant cases have been reported related syndromes in the previous literature. Our findings will help to broaden the clinical phenotypic spectrum of *IARS2* -related diseases. We considered that the *IARS2* variant may be related to the disruption of the immune barrier in the body which contributed to the infection in the proband.

## Conclusion

In summary, we report a compound heterozygous variant of *IARS2* gene. All published cases suggest that the variants of *IARS2* can cause a variety of clinical phenotypes. Its diagnosis is difficult due to the lack of typical clinical symptoms. If one relies solely on traditional biochemical and neuroimaging tests, *IARS2* -associated disease can easily be missed. In terms of disease diagnosis, WES is a powerful tool for the diagnosis of highly heterogeneous neurodevelopmental disorders. Genetic testing should be performed as early as possible if this disease is suspected. We believe that this case provides an important reference for the diagnosis and treatment of future cases.
